# Vacuolar-Iron-Transporter1-Like Proteins Mediate Iron Homeostasis in Arabidopsis

**DOI:** 10.1371/journal.pone.0110468

**Published:** 2014-10-31

**Authors:** Julia Gollhofer, Roman Timofeev, Ping Lan, Wolfgang Schmidt, Thomas J. Buckhout

**Affiliations:** 1 Institute of Biology, Humboldt University Berlin, Berlin, Germany; 2 State Key Laboratory of Soil and Sustainable Agriculture, Institute of Soil Science, Chinese Academy Sciences, Nanjing, PR China; 3 Academia Sinica, Institute of Plant and Microbial Biology, Taipei, Taiwan; 4 Graduate Institute of Biotechnology, National Chung Hsing University, Taichung, Taiwan; 5 Genome and Systems Biology Degree Program, College of Life Science, National Taiwan University, Taipei, Taiwan; Louisiana State University Health Sciences Center, United States of America

## Abstract

Iron deficiency is a nutritional problem in plants and reduces crop productivity, quality and yield. With the goal of improving the iron (Fe) storage properties of plants, we have investigated the function of three Arabidopsis proteins with homology to Vacuolar Iron Transporter1 (AtVIT1). Heterologous expression of *Vacuolar Iron Transporter-Like1* (*AtVTL1*; At1g21140), *AtVTL2* (At1g76800) or *AtVTL5* (At3g25190) in the yeast vacuolar Fe transport mutant, *Δccc1*, restored growth in the presence of 4 mM Fe. Isolated vacuoles from yeast expressing either of the *VTL* genes in the *Δccc1* background had a three- to four-fold increase in Fe concentration compared to vacuoles isolated from the untransformed mutant. Transiently expressed GFP-tagged AtVTL1 was localized exclusively and AtVTL2 was localized primarily to the vacuolar membrane of onion epidermis cells. Seedling root growth of the Arabidopsis *nramp3/nramp4* and *vit1-1* mutants was decreased compared to the wild type when seedlings were grown under Fe deficiency. When expressed under the 35S promoter in the *nramp3/nramp4* or *vit1-1* backgrounds, *AtVTL1*, *AtVTL2* or *AtVTL5* restored root growth in both mutants. The seed Fe concentration in the *nramp3/nramp4* mutant overexpressing *AtVTL1*, *AtVTL2* or *AtVTL5* was between 50 and 60% higher than in non-transformed double mutants or wild-type plants. We conclude that the VTL proteins catalyze Fe transport into vacuoles and thus contribute to the regulation of Fe homeostasis *in planta*.

## Introduction

Regulation of the cellular Fe concentration poses the problem of balancing Fe deficiency against toxicity [1]. Equilibrium is maintained by strict regulation of Fe uptake and storage in the cell. Uptake of soil Fe in Arabidopsis is catalyzed by the Fe^2+^ transporter AtIRT1 [2], [3]. Transcription of *AtIRT1* is greatly increased under conditions of Fe deficiency [2], and AtIRT1 is also post-translationally modified to regulate its partitioning between the trans-Golgi network and plasma membranes [4]. Prior to transport, soil Fe^3+^-chelates are reduced by the plasma membrane ferric reductase, AtFRO2 [5]. The coupling of uptake to reduction is a hallmark of strategy I plants, and an analogous mechanism is also found in *Chlamydomonas* [6] and *Saccharomyces* [7] among other organisms. The reduction-based acquisition of Fe is aided by secretion of Fe-binding compounds and H^+^-ATPase-mediated acidification of the rhizosphere [8], [9], [10], [11].

Once in the cell, Fe^2+^ can be incorporated into proteins or stored in cellular compartments. In chloroplast and presumably mitochondria, several thousand Fe atoms are stored per ferritin molecule [12]. However, in Arabidopsis only 5% of Fe is stored in ferritin, and vacuoles appear to be the major compartment for seed Fe storage [13]. Vacuolar Fe uptake was shown to be catalyzed by the ferroportin homologue, AtFPN2 [14], [15], and by the CCC1-like protein AtVIT1 (16); however, the primary substrates of AtFPN2 have been reported to be Ni and Co in addition to Fe [14], [15]. AtVIT1 has a specific function in the vacuolar transport of Fe into xylem parenchyma of developing embryos, and the *vit1-1* mutant shows misdistribution of Fe in seeds; although, *vit1-1* mutant seeds have unchanged Fe concentration compared with the wild type [16]. Efflux of Fe from the vacuole is catalyzed by two NRAMP proteins, NRAMP3 and NRAMP4. The double mutant *nramp3/nramp4* shows decreased Fe mobilization from the vacuole in germinating seeds [17].

Two rice orthologs of AtVIT1, OsVIT1 and OsVIT2 [18], and one in tulip, TgVIT1 [19], have been shown to catalyze vacuolar Fe transport. TgVIT1 catalyzes the transport of Fe into the proximal perianth cell vacuole, which was shown to be essential for blue color development in tulips. Both OsVIT1 and OsVIT2 complemented the yeast *Δccc1* mutant, and vacuoles that were isolated from complemented cells had increased Fe and Mn concentrations. In addition, both rice genes complemented the Zn transport mutant, *Δzrc1*, and vacuoles isolated from these cells also had an increased Zn concentration [18]. The transcript abundance of *OsVIT2* increased within hours in roots and shoots grown under conditions of excess Fe (4 mM) but decreased in roots and shoots when rice was grown under Fe-deficient conditions. In contrast, *OsVIT1* expression responded only weakly to changes in the Fe status. *OsVIT1* and *OsVIT2* were expressed in flag leaf blades and sheaths, respectively. Consistent with the localization of expression, the *osvit1-1* and *osvit2-1* T-DNA knockout mutants had decreased Fe and Zn content in flag leaves with no change in Mn. Seeds of *osvit1-1* and *osvit2-1* had correspondingly increased Fe and Zn content. OsVIT1 and OsVIT2 were shown to regulate the partitioning of Fe and Zn in rice between source and sink tissues [18].

In an analysis of the transcriptional response of Arabidopsis roots to Fe deficiency, we and others have identified three genes whose mRNA abundance decreased in Fe-deficient roots and whose putative amino acid sequences showed significant homology to AtVIT1 and consequently also to yeast Ccc1p [20], [21], [22]. These genes belong to a small, five-membered family that has been annotated as nodulin or nodulin-like in databases. These genes will be subsequently referred to as *Vacuolar Iron Transporter-Like* (*VTL*). The *VTL* family was found both in mono- and dicotyledon plants, as well as *Chamydomonas* and *Physcomitrella*. Promoter-β-glucuronidase (GUS) assays showed expression of *AtVTL1* in roots, hypocotyls, and cotyledons of seedlings with the greatest activity associated with the vascular bundle and the root stele [21]. The promoter activity was greatly reduced in Fe-deficient seedlings, consistent with the transcriptional analysis. In the present report, we show that AtVTL1 (At1g21140) and AtVTL2 (At1g76800) are localized to the vacuolar membrane in plants, and that AtVTL1, AtVTL2 and AtVTL5 (At3g25190) complement the *Δccc1* mutation in *Saccharomyces*. Over-expression of *AtVTL1*, *AtVTL2* and *AtVTL5* also complemented the Fe deficiency-dependent root growth phenotype in the *nramp3/nramp4* double mutation and the *vit1-1* mutation in Arabidopsis seedlings. These results indicate that the three members of the *VTL* family are involved in regulation of cellular Fe homeostasis, likely by acting as vacuolar Fe transporters.

## Materials and Methods

### Arabidopsis Growth Conditions

Arabidopsis seeds were surface-sterilized, vernalized and grown hydroponically as described by Gollhofer et al. [21]. Seeds of the accession Columbia (Col-0) were obtained from the Arabidopsis Biological Resource Center (Ohio State University, Columbus, Ohio). Plants were grown hydroponically according to the method described by Buckhout et al. [20], and the hydroponic nutrient solution was composed of KNO_3_ (3 mM), MgSO_4_ (0.5 mM), CaCl_2_ (1.5 mM), K_2_SO_4_ (1.5 mM), NaH_2_PO_4_ (1.5 mM), H_3_BO_3_ (25 µM), MnCl_2_ (1 µM), ZnSO_4_ (0.5 µM), (NH_4_)_6_Mo_7_O_24_ (0.05 µM) CuSO_4_ (0.3 µM), Fe-EDTA (40 µM) with the pH adjusted to 6.0 with KOH. For growth on Petri dishes, the method of Santi and Schmidt [11] was used. The medium contained: KNO_3_ (5 mM), Ca(NO_3_)_2_ (2 mM), MgSO_4_ (2 mM), KH_2_PO_4_ (2.5 mM), MnCl_2_ (14 µM), H_3_BO_3_ (70 µM), ZnSO_4_ (1 µM), CuSO_4_ (0.5 µM), Na_2_MoO_4_ (0.2 µM), CoCl_2_ (0.01 µM), NaCl (10 µM), and Fe-EDTA (40 µM). Sucrose (44 mM) and 5 mM MES (2-[N-morpholino]ethanesulfonic acid) were included, and the pH was adjusted to 5.5. The medium was solidified with 0.8% agar (Fluka, Taufkirchen, Germany). For determination of root growth seedlings were photographed at the appropriate time, the photographs enlarged and root length measured.

### Analysis of the Fe Concentration in Arabidopsis Seeds and in Yeast Vacuoles

The Fe content in seeds was determined by the BPDS method [23]. Dried and powdered samples (4–8 mg) were mixed with glass beads (∼20; ø 425–600 µm) in 2 ml Eppendorf tubes and incubated at 95°C in 75 µl nitric acid (65%) for 6 h to digest the plant material. Fifty µl of H_2_O_2_ (30%) was added and the solution incubated at 56°C for 2 h. The volume was adjusted to 200 µl with water. Twenty µl of this solution were diluted in 980 µl of BPDS buffer (1 mM bathophenanthroline disulfonic acid, 0.6 M sodium acetate and 0.48 M hydroxylammonium chloride). The concentration of Fe-BPDS was determined photometrically at 535 nm. A standard calibration curve was prepared by dilution of a stock FeSO_4_ solution dissolved in 0.1 N HCl. Samples were measured in triplicate and the experiments were conducted at least three times.

### Yeast Growth, Complementation and Isolation of Vacuoles

The yeast strain used in this study was *Δccc1* (*ura3, leu2, his3, ade2, can1, CCC1::HIS3*) in DY150. Yeast transformation was carried out by the Li-acetate method [24]. Yeast growth was determined under selection on agar plates with 10^0^–10^5^ cells per dot.

Vacuoles were isolated as described by Li et al. [25]. Briefly, cells harvest at OD_600_ = 0.4 were suspended in 30 ml of 0.1 M Tris-HCl (pH 9.3) buffer containing 10 mM dithiothreitol and incubated for 10 min at 30°C. The cells were washed once with spheroplast buffer (1.2 M sorbitol, 20 mM K-phosphate, pH 7.4) and incubated with 20 µg/ml lyticase for 30 min at 30°C. Spheroplasts were collected by centrifugation at 3,500 *g* for 5 min., and the pellet was resuspended in 10 ml of 15% Ficoll buffer (15% Ficoll (Sigma) in 0.2 M sorbitol and 10 mM PIPES-KOH, pH 6.8). Fifty µg/ml DEAE-Dextran (Amersham Pharmacia Biotech) were added to the spheroplasts, and the sample was incubated for 3 min. on ice and for an additional 5 min. at 30°C. The lysate (10 ml) was transferred to SW28 centrifuge tubes (Beckman Instruments) and overlaid with 10 ml of 8% Ficoll, 10 ml of 4% Ficoll, and 10 ml of 0% Ficoll. The tubes were centrifuged at 110,000 *g* for 3 h. The vacuolar fraction was collected from the 0%/4% interphase. Fe concentration of yeast vacuoles was determined by the BPDS method [23].

### Transformation and Transient Expression of *GFP::VTL*

Arabidopsis wild-type Col-0 was transformed using the floral dip method of Clough and Bent [26]. Transgenic plants were selected for BASTA resistance on potting soil.

Onion epidermal cells were co-bombarded with 2.5 µg of plasmids bearing free GFP or GFP-tagged *VTL* genes (35S:*GFP::AtVTL1* or 35S:*GFP::AtVTL2*) and the vacuole marker plasmid vac-rk CD3 975 [27]. Plasmids were coated on 1 µm gold particles and delivered into onion epidermal cells at a pressure of 900 psi by a PDS 1000/He particle delivery system (BioRad, U.S.A). After bombardment, onion slices (2 cm^2^) were placed in a Petri dish containing Murashige and Skoog (MS) salts, 30 g/l sucrose and 1.5% agar (pH = 5.7). Following a minimum of 24 h, the epidermis cells were observed under a confocal laser scanning microscope (Zeiss LSM510 Meta) using a ×63 water objective. Vac-rk CD3 975 images were captured in the 560 to 615 nm range after excitation at 543 nm with a HeNe laser beam. The GFP images were captured in the 505 to 530 nm range after excitation at 488 nm with an argon laser beam. Image overlay was carried out by Z-stack analysis at 0.8 µm intervals and further processed with the projection function under LSM510-Expert Mode software.

### qRT-PCR

Total RNA was isolated using TRIsure (Bioline) and treated with DNase using DNAse Kit (Fermentas) as suggested by the manufacturer. cDNA was synthesized using DNA-free RNA with oligo-dT(18) primer and RevertAid reverse transcriptase (Thermo scientific). The cDNA was used as a PCR template in a 10 µl reaction system using the SensiMix SYBR No-ROX Kit (Bioline) with programs recommended by the manufacturer in a CFX96 Realtime system (BioRAD). Three biological replicates were performed for each sample. The ΔΔC_T_ method was used to determine the relative transcript abundance

## Results

### Complementation of the yeast mutant *Δccc1* by *AtVTL1*, *AtVTL2* and *AtVTL5*

**Figure 1:**
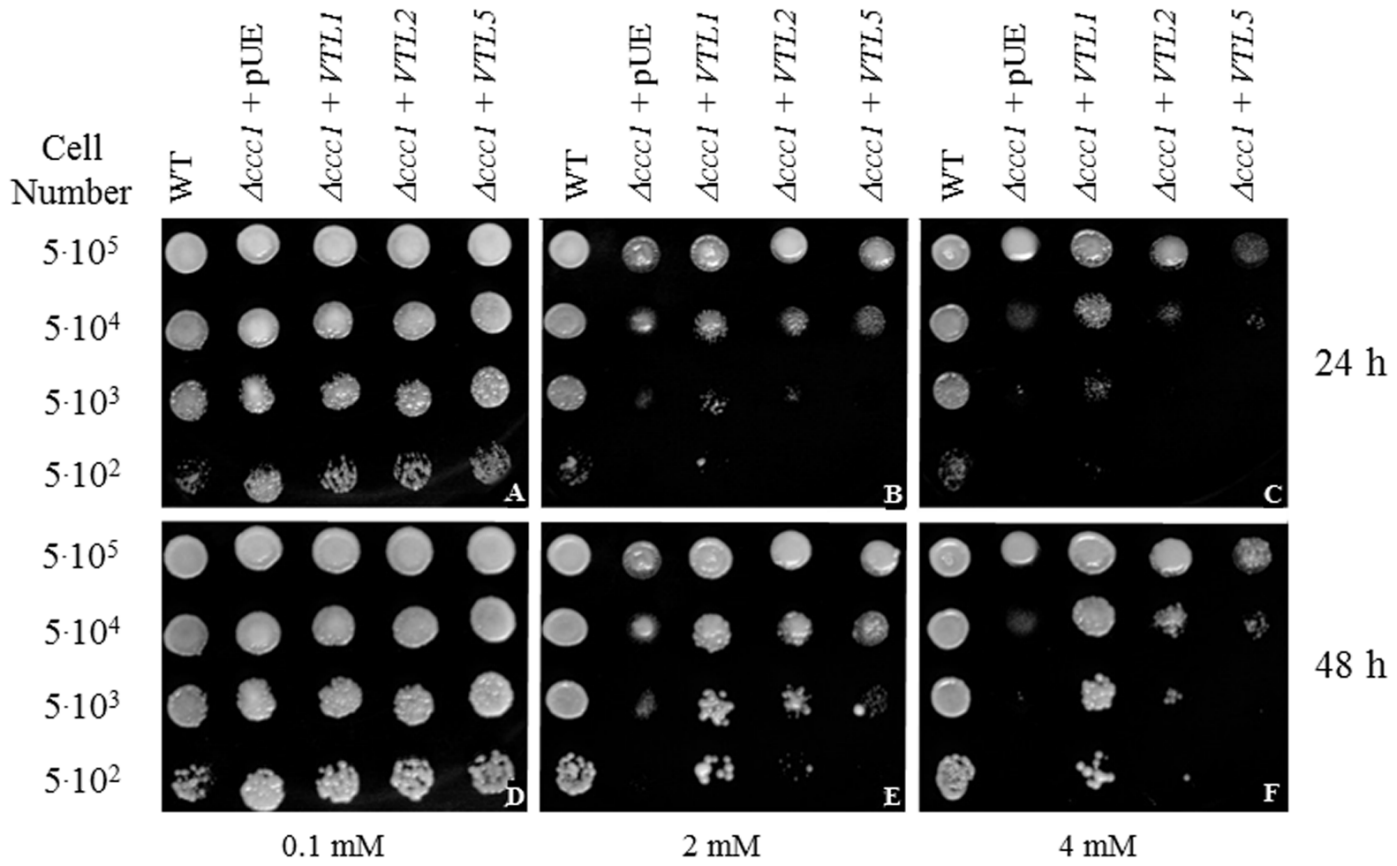
Complementation of the *Δccc1* by heterologous expression of the *VTL* genes. *Δccc1* (vacuolar Fe^2+^/Mn^2+^ transporter) cells were transformed with each of the three VTL genes or the empty vector (pUE) under the control of the PGK promoter and grown on SD medium containing FeSO_4_ at the concentrations indicated for 24 or 48 h at 30°C. Cells were plated at the densities indicated in the figure.

As in Arabidopsis, the yeast vacuole is the major compartment for Fe storage but also protects the cell from Fe toxicity. The yeast gene *CCC1* encodes a vacuolar Fe^2+^/Mn^2+^ transporter that catalyzes Fe^2+^ uptake into the vacuole. The *Δccc1* mutant is hypersensitive to Fe toxicity, not growing in the presence of greater than ca. 3 mM Fe [25]. Because of the homology of the VTL family member to yeast Ccc1p and Arabidopsis AtVIT1 and because of the transcriptional repression of *AtVTL1*, *AtVTL2* and *AtVTL5* under conditions of Fe deficiency, the ability of the three *AtVTL* genes to complement the *Δccc1* yeast mutant was investigated. When expressed in yeast, the VTL proteins were effective in complementing of the *Δccc1* phenotype in the order *AtVTL1*> *AtVTL2*> *AtVTL5* (Fig. 1). These results are consistent with an AtVTL-dependent decreased the cytosolic Fe in the *Δccc1* mutant, presumably by transport of Fe into the vacuole.

**Figure 2:**
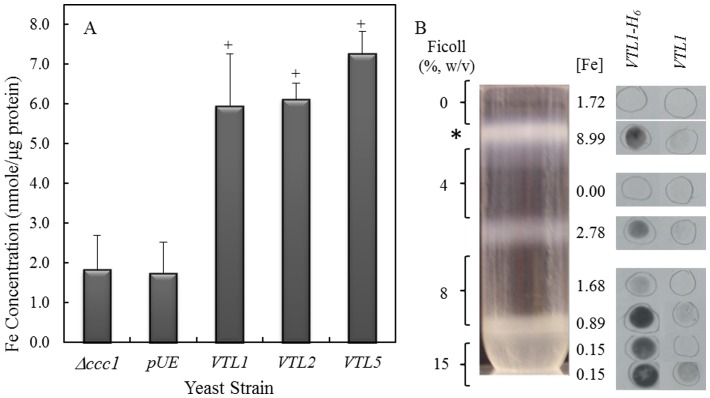
Determination of the Fe concentration in *Δccc1* cells transformed with *AtVTL1, AtVTL2* or *AtVTL5*. (**A**) Shown is the Fe concentration in yeast vacuoles isolated from the *Δccc1* mutant, *Δccc1* transformed with an empty plasmid (pUE), *AtVTL1, AtVTL2* or *AtVTL5*. Error bars indicate the standard error of the mean. Bar graphs labeled with “+” are significantly different for the *Δccc1* mutant (*p*<0.05). (**B**) Vacuoles were isolated from *Δccc1* yeast cells transformed with His-tagged *AtVTL1* (*VTL1-H_6_*) or with untagged *AtVTL1* (*VTL1*) as a control. AtVTL1-H_6_ was localized by dot-blots using an anti-His antibody. The positions of the dot-blots are approximate. The Fe concentrations (nmole/µg protein) in the fraction corresponding to the dot-blot are given.

To test this hypothesis, the Fe concentration in isolated yeast vacuoles was investigated. Intact yeast vacuoles were isolated by floatation through a Ficoll step gradient as described by Li et al. [25]. We confirmed the composition of the vacuolar fraction by re-centrifugation the fraction on a continuous sucrose gradient followed by marker enzyme analysis (Fig. S1). As expected, the vacuole marker, bafilomycin-sensitive ATPase, co-localized with membrane proteins and no detectable plasma membrane (vanadate-sensitive ATPase) or endoplasmic reticulum (NADPH cytochrome c reductase) activities were detected in the gradient. Vacuoles, isolated from log-phase *Δccc1* cells or from *Δccc1* cells transformed with the control vector grown in liquid SD medium containing 1 mM FeSO_4_, had approximately 2 nmoles Fe per µg protein, whereas *Δccc1* cells expressing *AtVTL1*, *AtVTL2* or *AtVTL5* had a greater than 3-fold higher Fe content (Fig. 2A).

Localization of AtVTL1 was demonstrated by transforming *Δccc1* cells with *AtVTL1* fused to a 3′ His tag (*AtVTL1*-*H_6_*). *Δccc1* cells that were transformed with *AtVTL1-H_6_* complemented the Fe-sensitive phenotype (Fig. S2). Using a His-tag antibody, AtVTL1-H_6_ protein was localized to the vacuolar fraction at the 0/4% Ficoll interface (Fig. 2B). Iron was also concentrated in this fraction compared to fractions in other regions of the Ficoll gradient. The immunological signals in the pellet and 15%/8% Ficoll interface were most like the result of vacuolar membranes from ruptured vacuoles that that did not float through the gradient. We demonstrated that AtVTL1 was localized on the yeast vacuolar membrane, where it presumably catalyzed transport of Fe into the yeast vacuole.

### Localization of AtVTL1 and AtVTL2 *in planta*

**Figure 3:**
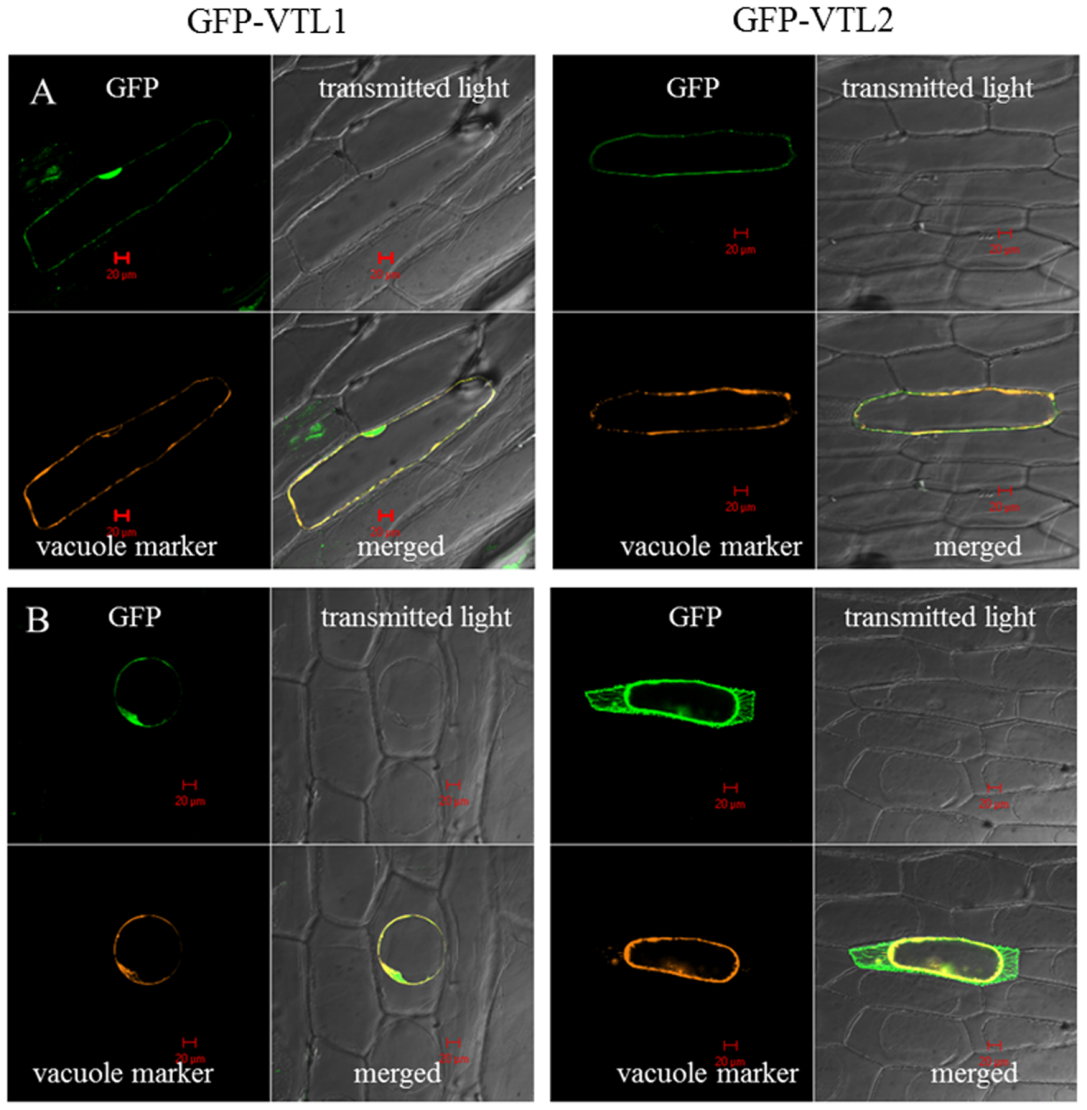
Transient 35S:GFP*::VTL1* and 35S:GFP*::VTL2* expression in onion epidermis cells. The GFP-VTL1 fluorescent signal co-localized with the vacuolar marker, vac-rk CD3-975, in turgescent (**A**) and plasmolyzed (**B**) cells. The GFP-VTL2 signal was also observed on the plasma membrane and cytoplasma (**B**).

The localization of AtVTL1 and AtVTL2 was investigated in onion epidermis cells. Attempts to localize AtVTL5 in onion or tobacco leaf cells have not been successful. Onion cells were transformed by particle bombardment with 35S:*GFP::VTL1* or 35S:*GFP::VTL2* reporter gene constructs. In the case of AtVTL1, the GFP fluorescence co-localized with the vacuolar marker vac-rk CD3-975 (Fig. 3A, [27]). When cells were plasmolyzed in 0.8 M mannitol, the GFP signal remained associated with the vacuole (Fig. 3B). The localization of AtVTL2 in epidermis cells was predominately associated with the vacuolar membrane (Fig. 3A); however, GFP fluorescence was also found to be associated with the plasma membrane and the cytoplasm upon plasmolysis (Fig. 3B). The localization of the two AtVTL proteins on the onion vacuolar membrane was consistent with the complementation results in *Saccharomyces* and with the increased Fe concentration in vacuoles isolated from *VTL*-expressing yeast cells.

### Complementation of the *nramp3/nramp4* and the *vit1-1* mutants

**Figure 4:**
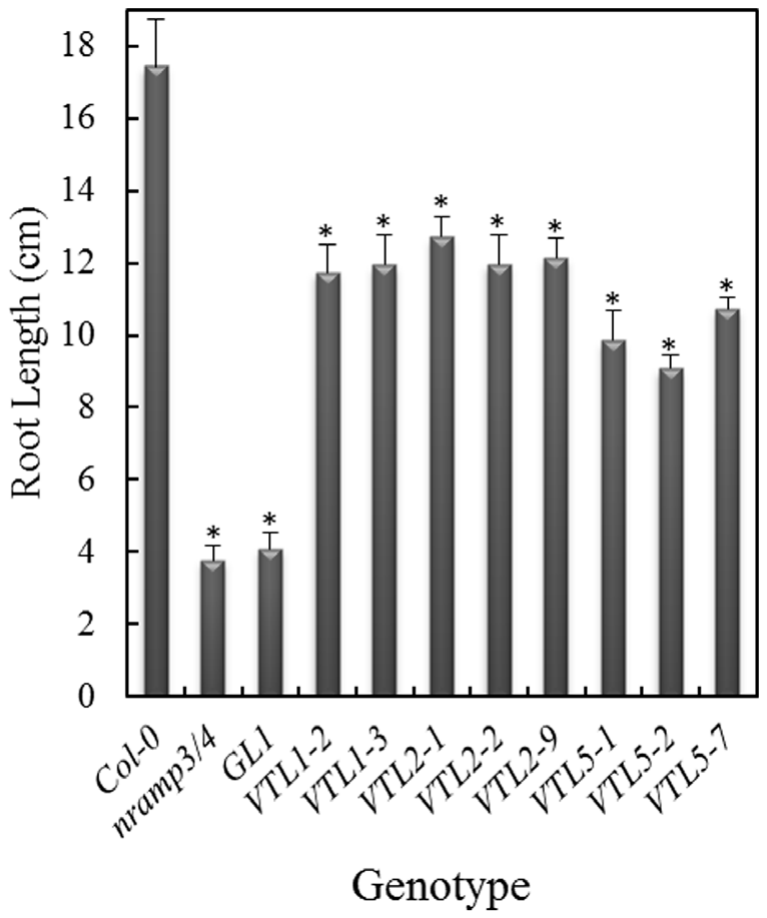
Complementation of the *nramp3/nramp4* double mutant by over-expression of AtVTLs. The three VTL genes were over-expressed in the *nramp3/nramp4* double mutant background under the control of the 35S promoter and root length was determined after 5 days of growth. Seedlings were grown without added Fe. Seedlings were grown on agarose Petri plates as described in the Materials and Methods. Asterisks indicate significant differences from the wild-type (p<0.001).

Over-expression of *AtVTL1* in Arabidopsis did not greatly alter Fe content of shoots and roots [21], nor were changes observed in the kinetics of the response to Fe deficiency, as determined by the root plasma membrane Fe^3+^-chelate reductase and chlorophyll content (Figs. S3A and B). In an attempt to characterize their functions, we have ectopically expressed the *AtVTL* genes in the Arabidopsis *nramp3/nramp4* double mutant background (17). Over-expression was confirmed by semi-quantitative RT-PCR (Fig. S4). Both NRAMP3 and NRAMP4 have been shown to be Fe vacuolar efflux carriers. The double mutant had no obvious phenotype when grown on soil; however, growth of mutant seedlings was retarded shortly after germination on an Fe-deficient substrate [17]. The phenotype was not persistent and disappeared after a few days of growth or with supplemental Fe in the media. Under Fe deficiency root growth of the *nramp3/nramp4* double mutant and of the double mutant transformed with the empty vector (GL1), was greatly inhibited compared to the wild-type control at 5 d following germination (Fig. 4 and Fig. S5; *p*<0.001) [17]. Importantly, over-expression of *AtVTL1*, *AtVTL2* or *AtVTL5* in *nramp3/nramp4* complemented this mutant growth phenotype. The early seedling root length was significantly increased (Fig. 4 and Fig. S5; *p*<0.001) compared to the double mutant; however, the roots were still significantly shorter than those of the wild type (*p*<0.001). Thus, over-expression of *AtVTL1*, *AtVTL2* or *AtVTL5* partially restored the wild-type phenotype in the *nramp3/nramp4* double mutant.

**Figure 5:**
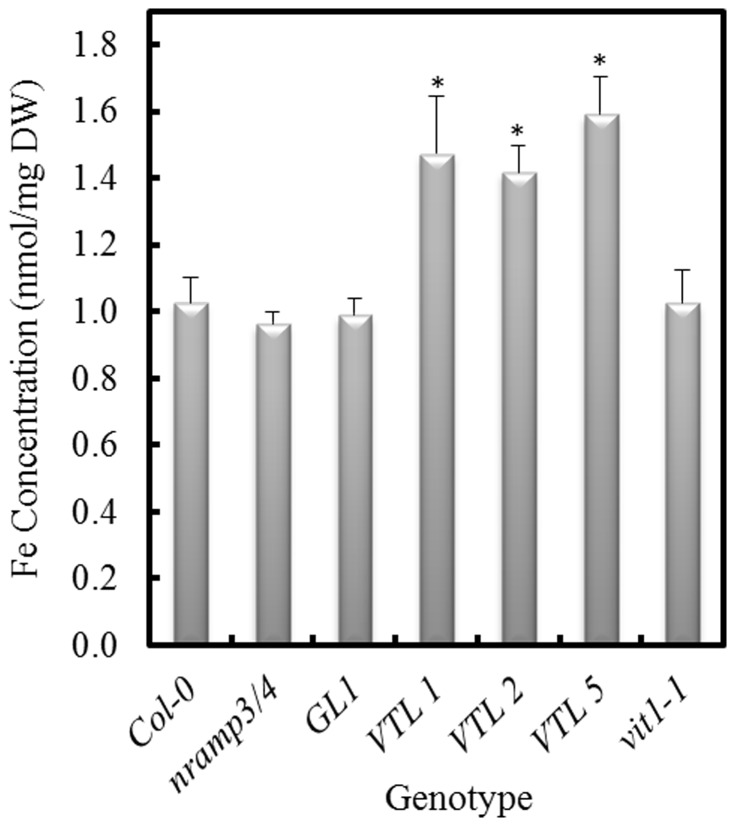
Fe concentration of Arabidopsis seeds. Fe was determined in the wild-type (Col-0), the *nramp3/nramp4* double mutant, the vector control (GL1), the *vit1-1* mutant and in seeds from the double mutant expressing one of the three VTL genes under the control of the 35S promoter. Asterisks indicate significant differences from the wild-type (p<0.01).

The proposed function of NRAMP3 and NRAMP4 was to mobilize Fe from the vacuole of young seedlings [17]. We hypothesized that over-expression of the AtVTLs might have increased total seed Fe, and thereby increased the Fe supply to the germinating seedling. To test this, we analyzed the Fe concentration in *nramp3/nramp4* seeds that over-expressed the *AtVTL* genes. The results of these analyses demonstrated that the *AtVTL1*, *AtVTL2* or *AtVTL5* over-expressing lines had between 40 and 60% increased Fe concentration compared to either the wild type or the *nramp3/nramp4* double mutant (Fig. 5; *p*<0.01). These results supported the concept that an increased content of Fe in the seeds could have supplied the embryo with sufficient Fe even when efflux from the vacuole through the NRAMP transporters was compromised.

**Figure 6:**
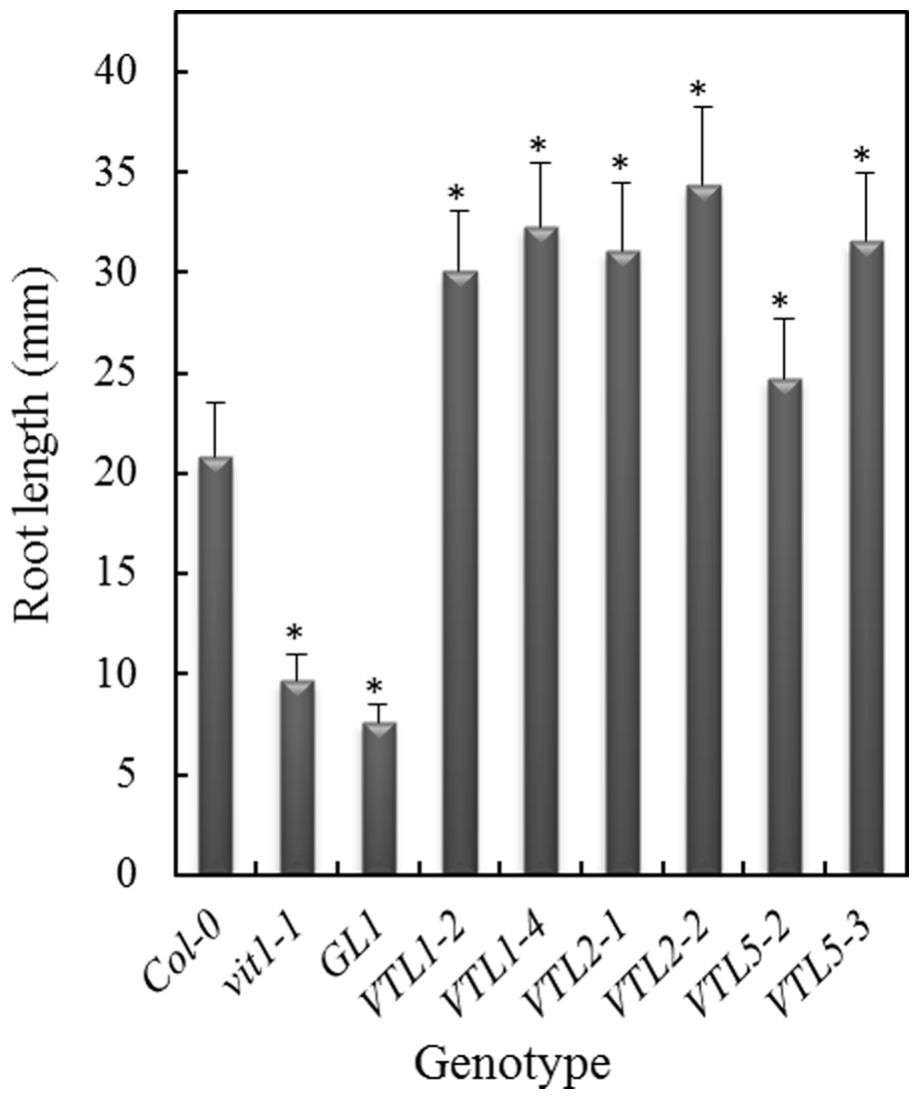
Complementation of the *vit1-1* mutant by over-expression of *AtVTL1*, *AtVTL2* or *AtVTL5*. The three VTL genes were over-expressed under the control of the 35S promoter in the *vit1-1* background. Root length was determined after 13 days of growth on agarose Petri plates as described in the Materials and Methods ion ES medium without added Fe and in the presence of the Fe^2+^ chelator, Ferrozine. Asterisks indicate significant differences from the wild-type (p<0.001). Shown are results from 2 independent transformants.

As mentioned above, the CCC1-like protein, AtVIT1, is localized on the vacuolar membrane and functions in the transport of Fe into the parenchyma cell of the provascular strands in developing embryos. The *vit1-1* mutant showed misdistribution of Fe; although, *vit1-1* mutant seeds had unchanged Fe content compared to the wild type (Fig. 5) [16]. *vit1-1* had a severely chlorotic phenotype when grown on alkaline soil [16]. In addition, we observed that *vit1-1* displayed a short-root phenotype similar to the *nramp3/nramp4* mutant when grown on Fe-deficient media in the presence of the Fe^2+^ chelator Ferrozine (Fig. 6 and Fig. S6). Over-expression of *AtVTL1*, *AtVTL2* or *AtVTL5* restored root growth to greater than the wild-type length (*p*>0.001), thus complementing the *vit1-1* mutation.

### Regulation of *AtVTL1*, *AtVTL2* and *AtVTL5* Gene Expression

**Figure 7:**
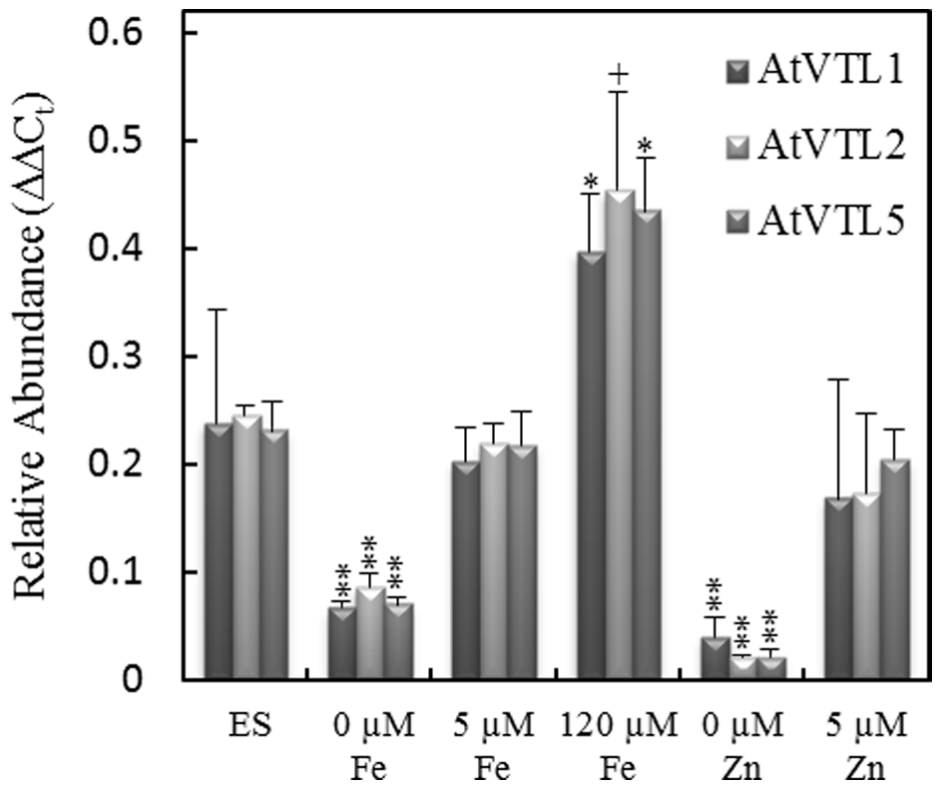
RT-qPCR of At*VTL1*, At*VTL2* and *AtVTL5* in response to Fe and Zn supply. Seedlings were grown on agarose petri plates as described in the Materials and Methods. Growth conditions were: ES medium (ES); “0”, ES without Fe or Zn or at the concentrations indicated. RT-qPCR was standardized to the expression of the *ACTIN2*. Symbols indicate significant differences to expression in the ES medium in three biological replicates: **, *p*<0.001; *, *p*<0.01; and +, *p*<0.05.

Finally, we investigated the response of *AtVTL1*, *AtVTL2* and *AtVTL5* to nutrient supply using quantitative qRT-PCR. Previously, we reported that the transcriptional activity of these genes positively correlated with the Fe supply [20]. Quantitative RT-PCR analyses confirmed these results (Fig. 7). Compared to Fe-sufficient controls (40 µM Fe), the transcript abundance of *AtVTL1*, *AtVTL2* and *AtVTL5* was decreased under Fe deficiency and increased in plants grown on media containing 120 µM Fe. Similarly, transcript abundance also decreased under Zn deficiency for *AtVTL1*, *AtVTL2* and *AtVTL5* (Fig. 7). However, when the Zn concentration was increased to 5 µM, the transcript abundance for *AtVTL1, AtVTL2* and *AtVTL5* was not significantly different from that of control plants grown on standard media (Fig. 7). The *VTL* genes were unable to restore growth in the Zn sensitive *Δzrc1* mutant (Fig. S7). Thus, a function of AtVTL1, AtVTL2 or AtVTL5 in vacuolar Zn transport is unlikely. Since *AtVTL1, AtVTL2* and *AtVTL5* responded positively to Fe supply and since their over-expression complemented vacuolar Fe transport mutations in yeast and Arabidopsis mutants, we conclude that *VTL* genes encode protein involved in Fe homeostasis, presumably as Fe vacuolar transporters.

## Discussion

Heterologous expression of *AtVIT1*, *OsVIT1, OsVIT2* and *TgVIT1* in the *Δccc1* mutant has been shown to complement the *Δccc1* mutation by restoring Fe uptake into the vacuole, and thus, to protect the cell from the deleterious effects of high cytosolic Fe ([6], [18], [19]. Vacuolar Fe transporter activity has been reported for FPN2, NRAMP3 and NRAMP4. Whereas AtVIT1 and FPN2 transported Fe into vacuoles, the two NRAMPs were shown to be efflux carriers. Previously, we have identified five genes in Arabidopsis, which encoded proteins with significant sequence homology to AtVIT1 [20]. The transcript abundance of *AtVTL1*, *AtVTL2* and *AtVTL5* was rapidly decreased in roots under conditions of Fe deficiency [21], [28]. We have shown in this report, that heterologous expression of *AtVTL1, AtVTL2* and *AtVTL5* in yeast *Δccc1* cells restored their ability to grow in the presence of 4 mM Fe. Since the heterologous expression of these genes in yeast correlated with increased Fe in the vacuolar fraction, we proposed that these three proteins increased Fe transport into the vacuole and thereby reduced toxic Fe in the cytoplasm of *Δccc1* cells.

### VTLs are Localized to the Arabidopsis Vacuolar Membrane

Transient expression of *GFP::VTL1* and *GFP::VTL2* chimeric proteins in onion epidermis cells resulted in a specific co-localization of GFP::VTL1 with the vacuolar marker vac-rk CD3-975. In the case of *GFP::VTL2* transient expression, a prominent signal was co-localized with the vacuole marker, but GFP fluorescence was also found in the cytoplasm and associated with the plasma membrane, indicating possible localization of AtVTL2 on other membranes in addition to the vacuole. Attempts at localizing AtVTL5 have not been successful.

Evidence for a function of AtVTL1, 2 and 5 in Fe homeostasis was obtained by complementation of the *nramp3/nramp4* and *vit1-1* mutations. The NRAMP3 and NRAMP4 proteins act redundantly as vacuolar Fe efflux carriers. The abundance of both NRAMPs was increased during Fe deficiency, and in the double mutant, greening of cotyledons and early root growth of seedlings were delayed compared to controls when seeds were germinated under Fe deficiency [17]. This inhibition of root growth could be largely eliminated by supplying Fe to the growth medium. The root growth phenotype was complemented by over-expression of *AtVTL1, 2* or *5* in the *nramp3/nramp4* mutant. In addition the total Fe content of seeds from over-expressing *AtVTL1*, *2* or *5* plants was increased compared to the wild type and the mutant. It might have seemed paradoxical that a mutation in a vacuole efflux transport system could be complemented by a putative vacuolar uptake transporter; however, an increased vacuolar Fe concentration might have been sufficient to compensate for a decreased efflux caused by the mutations in *NRAMP3* and *NRAMP4*.

The *vit1-1* mutant also displayed a Fe deficiency-dependent inhibition of seedling root growth that is likely the result of a mis-localization of Fe in the seed radicle. Over-expression of *AtVTL1, 2* or *5* might have restored the Fe supply to the growing root. In summary and based on the complementation activity in yeast, the increased vacuolar Fe concentration in yeast cells expressing AtVTL1, 2 or 5, the localization of AtVTL1 and 2 on the vacuolar membrane in onion epidermis cells and the restoration of seedling root growth in the *nramp3/nramp4* and *vit1-1* mutants, a critical role for the VTL proteins in the subcellular distribution of Fe is apparent.

### AtVTLs and Metal Homeostasis

Low substrate specificity is commonly found in cation transports. For example, AtIRT1 is the predominant plasma membrane Fe^2+^ transporter and is essential for Fe uptake from the soil [3]. In the absence of Fe or in the presence of excess divalent cations, AtIRT1 can also catalyze the transport of Zn, Mn and Cd [3], [29]. Both NRAMP3 and NRAMP4 have been shown also to be vacuolar Mn exporters [30]; although, transcription was correlated with the Fe and not the Mn nutrient status. Lanquar et al. [30] envision a passive function of NRAMP3 and NRAMP4 in cycling of Mn from the vacuole to the plastid in mesophyll cells. The *nramp3/nramp4* double mutant is hypersensitive to Zn and Cd, and as Sinclair and Krämer [31] suggested, the cause of the hypersensitivity appeared to be an indirect effect related to impaired Fe and Mn transport. Although the transcript data do support a role for the VTLs in Fe homeostasis, a role in homeostasis of other divalent cations analogous to the roles of NRAMP3 and NRAMP4 cannot be excluded.

Five Zn transport genes are among the Arabidopsis genes that response to Fe deficiency [32]. Four of these genes that transport Zn into the vacuole are strongly induced under Fe deficiency. The remaining gene, *AtZIP3*, was repressed and shown to catalyzed Zn uptake into cells. Zn has been shown to accumulate in roots grown under Fe-deficient conditions, likely the result of the Zn transport activity of IRT1 [3], [31]. It is noteworthy that the expression of *AtVTL1*, *AtVTL2* and *AtVTL5* was greatly decreased under Zn deficiency but unchanged when grown under Zn excess (Fig. 7). Expression of *AtVTL1*, *AtVTL2* and *AtVTL5* in the yeast *Δzrc1*, a vacuolar Zn transport mutant, did not complement this mutation (Fig. S7).

Although a direct role of the VTL proteins in Zn transport is lacking, an involvement of Zn in Fe homeostasis has been clearly demonstrated. Transcriptional regulation in strategy I plants to Fe deficiency is mediated in part by the bHLH transcription factors FIT (bHLH029) [33], [34], [35] and POPEYE (bHLH047, PYE) [36]. The central components of PYE regulation include PYE (At3g47640), IAA*–*LEUCINE-RESISTANT3 (ILR3, bHLH105, At5g54680) and BRUTUS (BTS, At3g47640). *PYE* is weakly and *BTS* is strongly induced under Fe deficiency [20], [36], and PYE and BTS both interact but with ILR3 in a yeast two-hybrid assay but not with each other [36]. Either a ternary complex of PYE-ILR3-BTS or the competition between PYE and BTS for ILR3 has been proposed as the regulatory switch in the PYE network [36]. PYE directly regulated genes that were associated with the response to Fe deficiency including: *FRO3*, *NAS4* and *ZIF1.* The transcription of *AtVTL1*, *2* and *5* was increased 1.9, 16.9 and 2.9-fold, respectively, in the *pye-1* knockout mutant [36], and roots of the *pye-1* mutant had an elevated Fe content compared with controls. These observations are consistent with the positive correlation between the root Fe concentration and transcript abundance of *AtVTL1*, *2* and *5* (Fig. 7) [21].

In the *ilr3-1* gain-of-function mutant, the transcript abundance of *AtVTL1*, *2* and *5* was decreased between 3- and 4-fold; an observation that can be explained by a gain-of-function in the PYE-ILR3-BTS repression [22]. In general, BTS and the rice homologs OsHRZs negatively regulate the Fe deficiency response in Arabidopsis and rice, and thus plants with decreased expression of these genes showed tolerance to Fe deficiency [36], [37]. Recombinant BTS has been shown to bind both Fe and Zn, primarily at the hemerythrin domain of the protein [36]. In a recent model, Kobayshi and Nishizawa [38] have proposed a mechanism for transcriptional regulation through the BTS/OsHRZ proteins based on binding of Zn and Fe to the hemerythrin domain of these proteins. In their model, Fe binding to BTS/OsHRZ in the absence of Zn would lead to repression PYE-regulated gene expression as seen in Fig. 7. The implications of their model are germane to the repression of *VTL* transcription under Zn deficiency observed in our experiments. Taken together, we have identified a group of putative Fe transporters that participate in the Fe homeostasis in Arabidopsis. As vacuolar Fe transporters these proteins may have importance in biofortification efforts in the future.

## Supporting Information

Figure S1 **Sucrose density gradient isolation of membrane in the yeast vacuolar fraction.** The vacuolar fraction was isolated as described in the Materials and Methods, and the vacuoles ruptured by repeated pipetting. The membranes were layered onto a continuous, 10 to 60% sucrose gradient and centrifuged at 110,000x*g* in a swing-out rotor over-night. The gradient was fractionated into 1 ml fractions and marker enzymes for the vacuole (bafilomycin-sensitive ATPase), plasma membrane (vanadate-sensitive ATPase) and endoplasmic reticulum (cytochrome *c* reductase) were determined by the method of Luster and Buckhout (Plant Physiol. 1989; 91(3): 1014-9). The activity of the vanadate-sensitive ATPase and the cytochrome c reductase were below the limits of detection.
(TIF)

Figure S2 **Complementation of the yeast** ***Δccc1*** **(vacuolar Fe^2+^/Mn^2+^ transporter) mutant with his-tagged** ***AtVTL1*** **and** ***AtVTL2*** **genes.** Cells were transformed with the empty vector (pUE) or the *VTL* gene containing a H_6_ tag under the control of the PGK promoter and grown on YPD medium containing 7.5 mM FeSO_4_.
(TIF)

Figure S3 **A. Chlorophyll content in wild-type (Col-0) and wild-type plants over-expressing** ***AtVTL1*** **grown in the Fe concentrations indicated.** Shown are chlorophyll a and b and the chlorophyll a/b ratio. **B. Analysis of the Fe^3+^-chelate reductase activity in Col-0 and Col-0 plants over-expressing** ***AtVTL1*** **.** Bars are standard error of the mean.
(TIF)

Figure S4 **Semi-quantitative PCR of of** ***AtVTL1*** **,** ***AtVTL2*** **and** ***AtVTL5*** **.** Expression was determined in Col-0 (WT), the *nramp3/nramp4* double mutant and in the double mutant over-expressing each of the *VTL1, VTL2* or *VTL5* genes. Expression was standardized to the level of *ACTIN2* expression.
(TIF)

Figure S5 **Root growth in the** ***nramp3/nramp4*** **double mutant transformed with** ***AtVTL1*** **,** ***AtVTL2*** **or** ***AtVTL5*** **.** Seedlings were grown for 5 days on standard media lacking Fe (see Materials and Methods). Shown are results from two to three independent transformants taken from one repetition of the experiment reported in Fig. 4.
(TIF)

Figure S6 **Root growth in the** ***vit1-1*** **mutant transformed with** ***AtVTL1*** **,** ***AtVTL2*** **or** ***AtVTL5*** **.** Seedlings were grown for 13 days on standard media (see Materials and Methods) lacking Fe and in the presence of the Fe^2+^ chelator, Ferrozine. Shown are results from an experiment similar to that reported in Fig. 6.
(TIF)

Figure S7 **Complementation of the** ***Δzrc1*** **by heterologous expression of the** ***VTL*** **genes.** *Δzrc1* (vacuolar Zn^2+^ transporter) cells were transformed with each of the three VTL genes or the empty vector (pUE) under the control of the PGK promoter and grown on SD medium containing ZnSO_4_ at the concentrations indicated for 24 or 48 h at 30°C. Cells were plated at the densities indicated in the figure.
(TIF)
