# Supplementary figures and images for: Vacuolar-Iron-Transporter1-Like Proteins Mediate Iron Homeostasis in Arabidopsis
Source: PLoS One. 2014 Oct 31;9(10):e110468. doi: 10.1371/journal.pone.0110468 (PMC4215979; doi:10.1371/journal.pone.0110468)

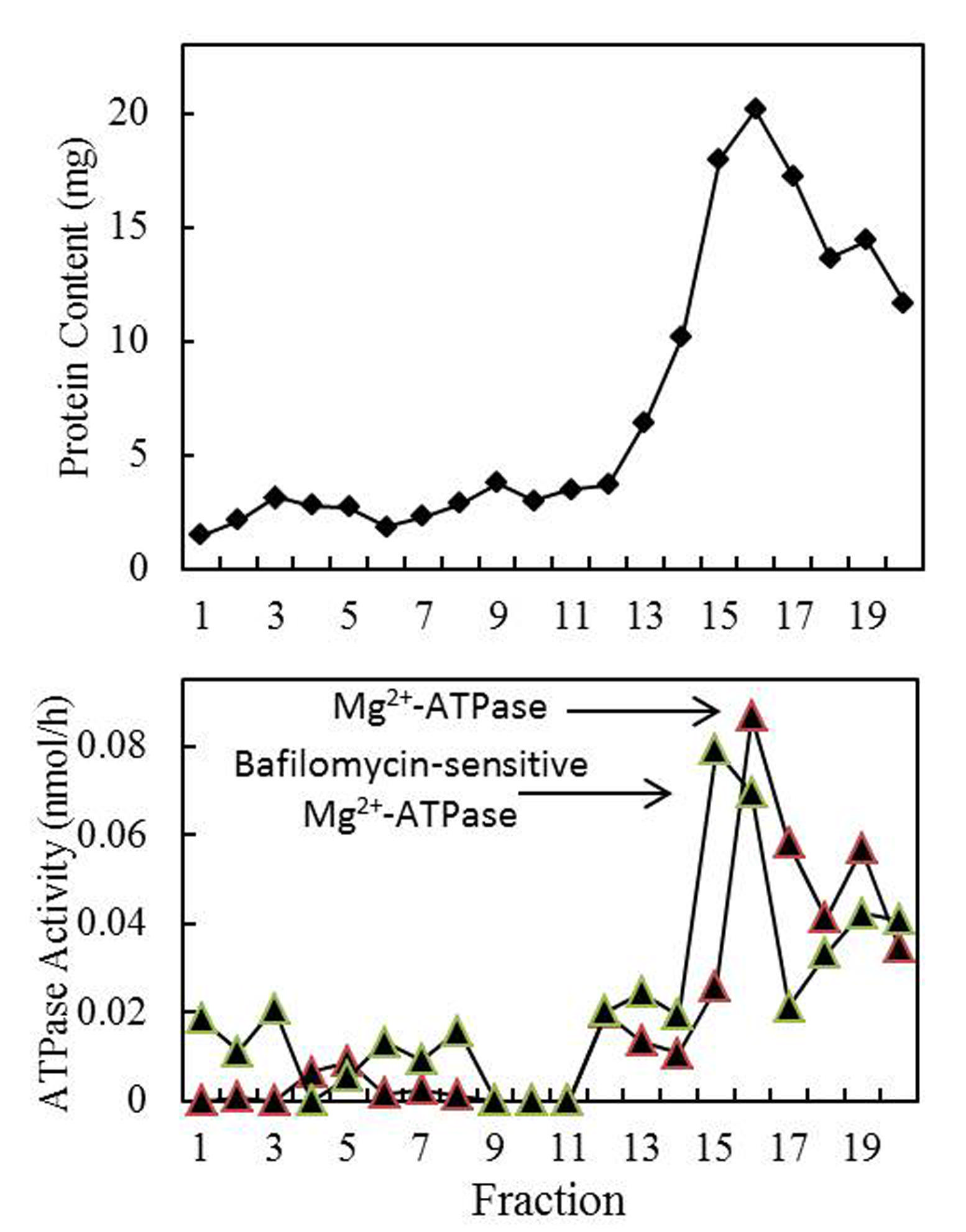

Supplement: Figure S1 — Sucrose density gradient isolation of membrane in the yeast vacuolar fraction. The vacuolar fraction was isolated as described in the Materials and Methods, and the vacuoles ruptured by repeated pipetting. The membranes were layered onto a continuous, 10 to 60% sucrose gradient and centrifuged at 110,000xg in a swing-out rotor over-night. The gradient was fractionated into 1 ml fractions and marker enzymes for the vacuole (bafilomycin-sensitive ATPase), plasma membrane (vanadate-sensitive ATPase) and endoplasmic reticulum (cytochrome c reductase) were determined by the method of Luster and Buckhout (Plant Physiol. 1989; 91(3): 1014-9). The activity of the vanadate-sensitive ATPase and the cytochrome c reductase were below the limits of detection. (TIF) [file pone.0110468.s001.tif]

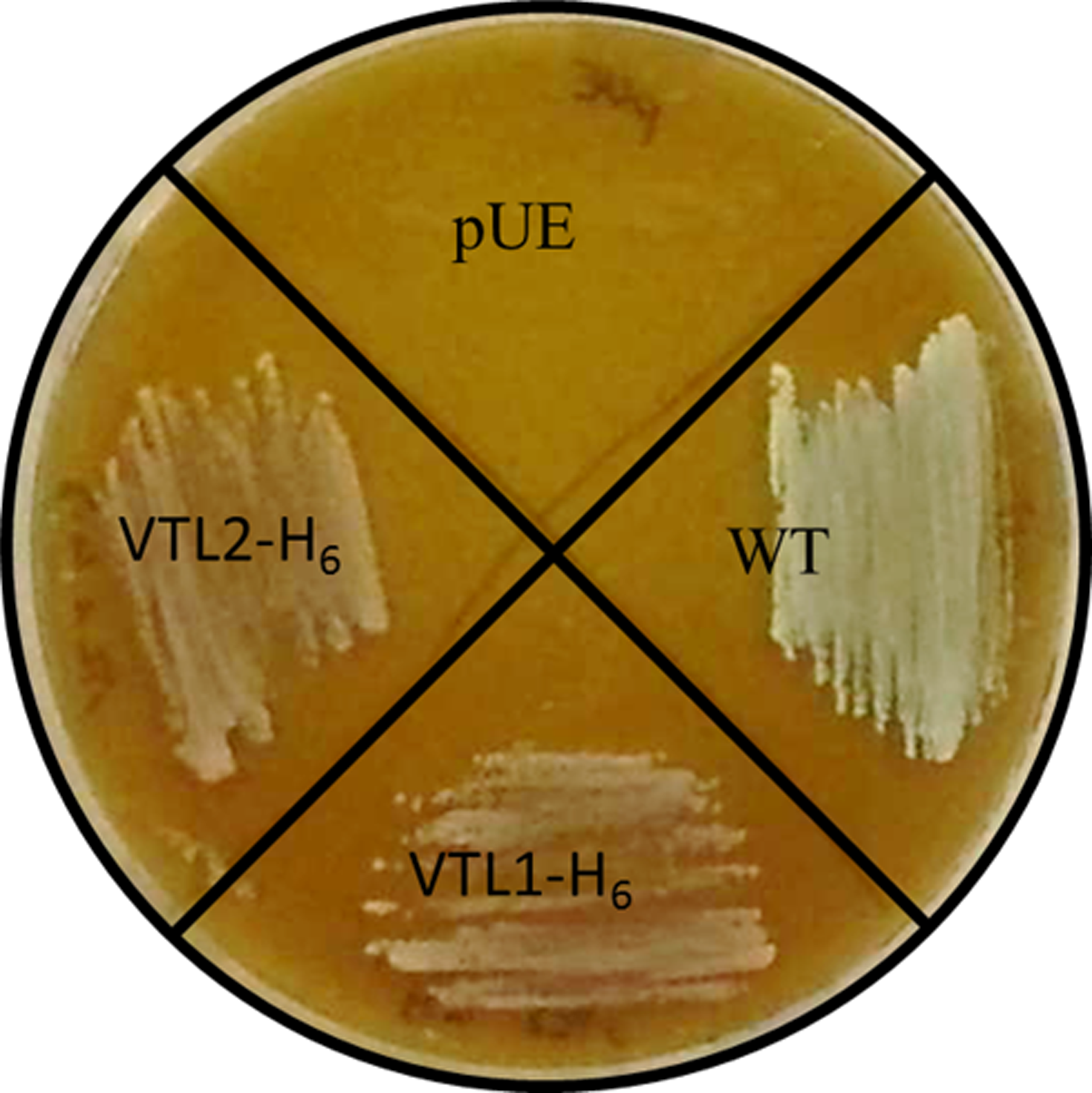

Supplement: Figure S2 — Complementation of the yeast Δccc1 (vacuolar Fe2+/Mn2+ transporter) mutant with his-tagged AtVTL1 and AtVTL2 genes. Cells were transformed with the empty vector (pUE) or the VTL gene containing a H6 tag under the control of the PGK promoter and grown on YPD medium containing 7.5 mM FeSO4. (TIF) [file pone.0110468.s002.tif]

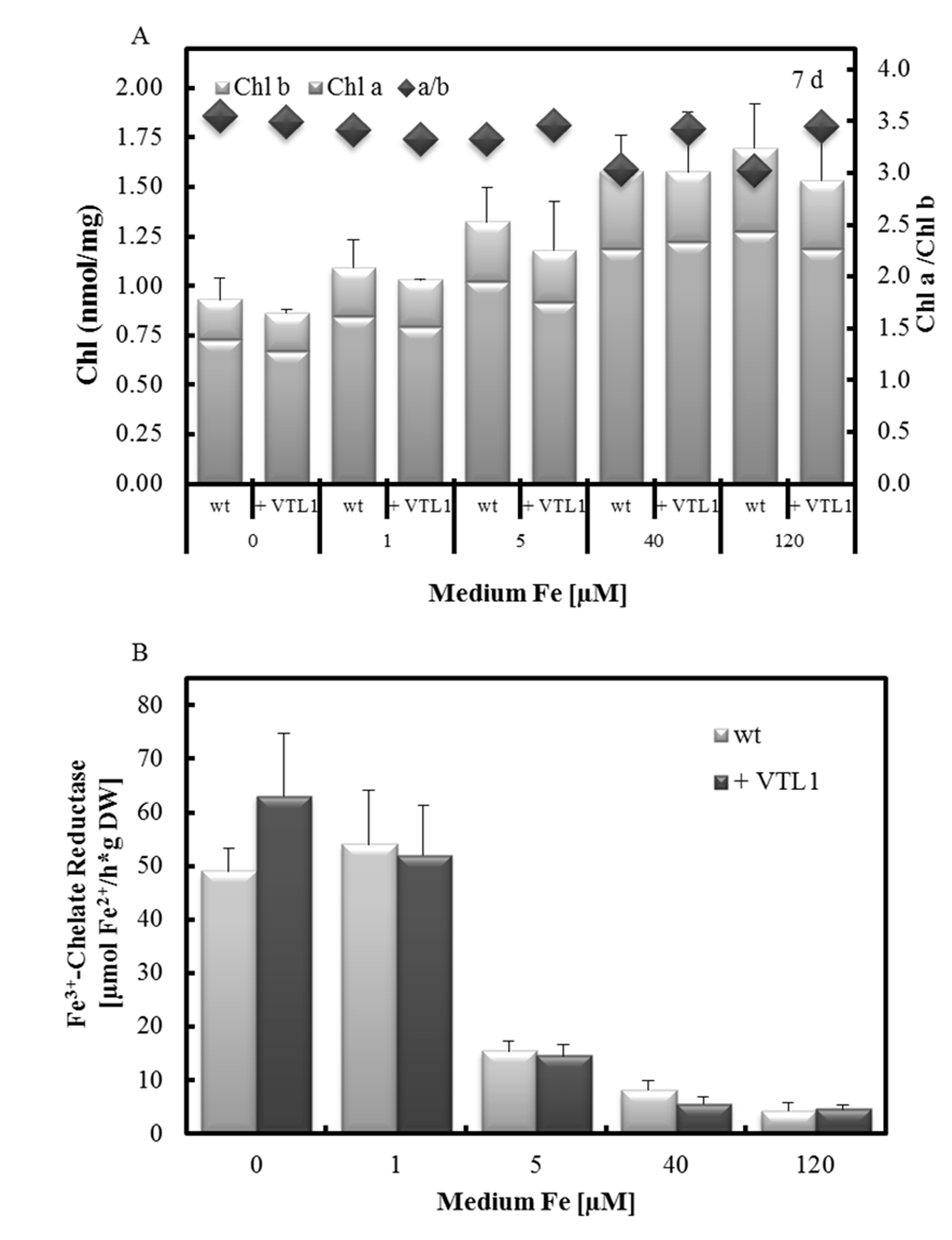

Supplement: Figure S3 — A. Chlorophyll content in wild-type (Col-0) and wild-type plants over-expressing AtVTL1 grown in the Fe concentrations indicated. Shown are chlorophyll a and b and the chlorophyll a/b ratio. B. Analysis of the Fe3+-chelate reductase activity in Col-0 and Col-0 plants over-expressing AtVTL1 . Bars are standard error of the mean. (TIF) [file pone.0110468.s003.tif]

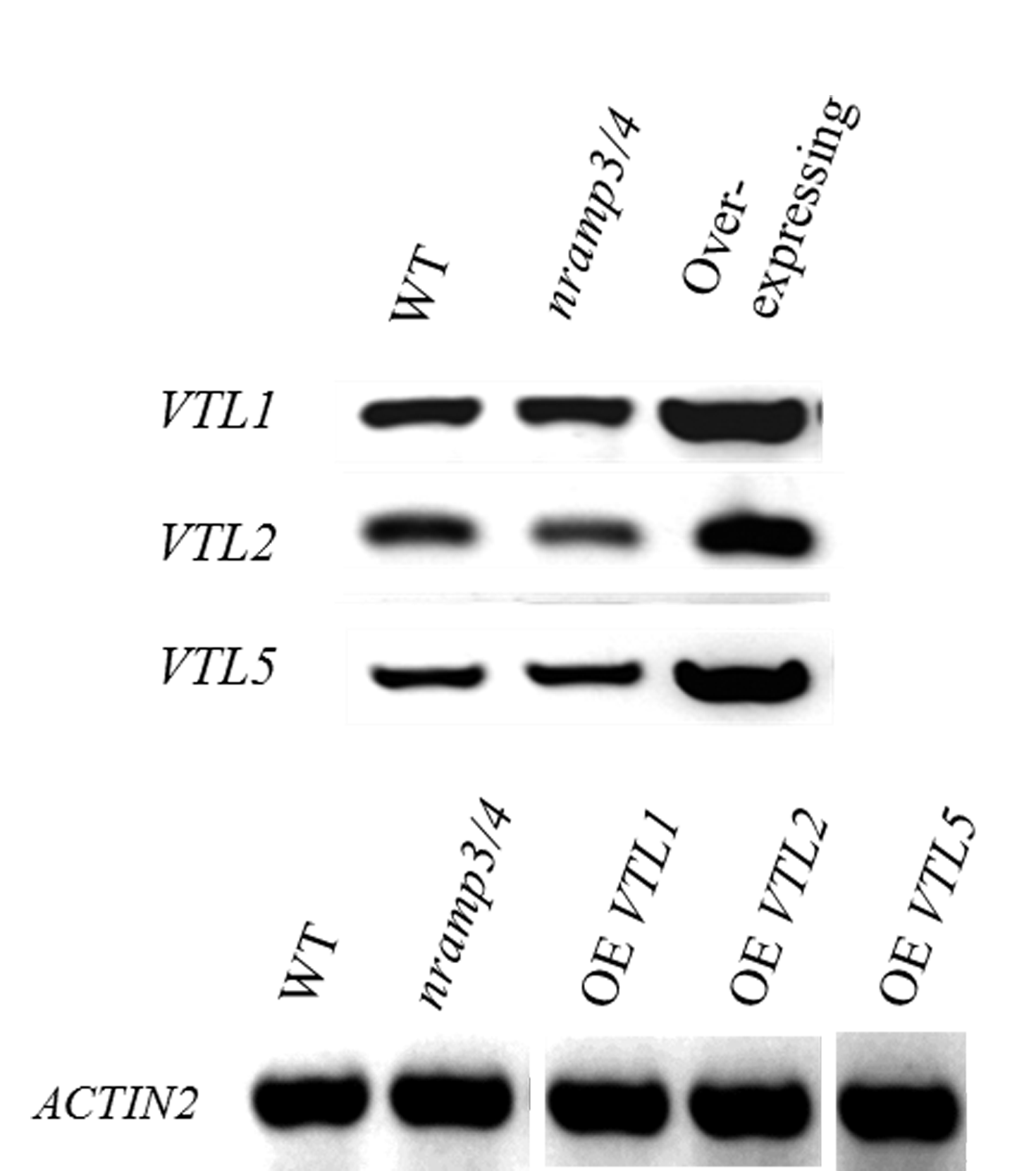

Supplement: Figure S4 — Semi-quantitative PCR of of AtVTL1 , AtVTL2 and AtVTL5 . Expression was determined in Col-0 (WT), the nramp3/nramp4 double mutant and in the double mutant over-expressing each of the VTL1, VTL2 or VTL5 genes. Expression was standardized to the level of ACTIN2 expression. (TIF) [file pone.0110468.s004.tif]

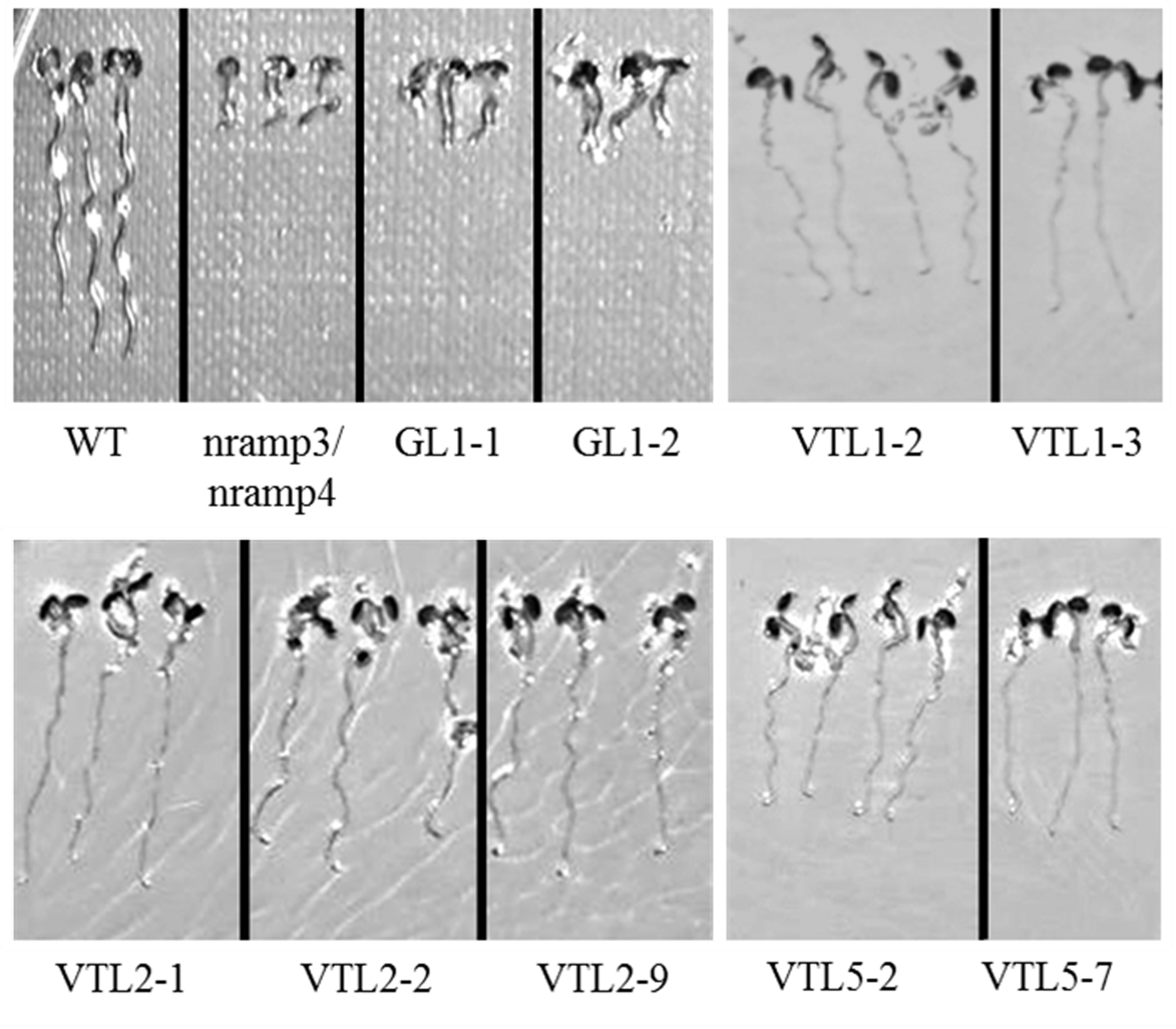

Supplement: Figure S5 — Root growth in the nramp3/nramp4 double mutant transformed with AtVTL1 , AtVTL2 or AtVTL5 . Seedlings were grown for 5 days on standard media lacking Fe (see Materials and Methods). Shown are results from two to three independent transformants taken from one repetition of the experiment reported in Fig. 4. (TIF) [file pone.0110468.s005.tif]

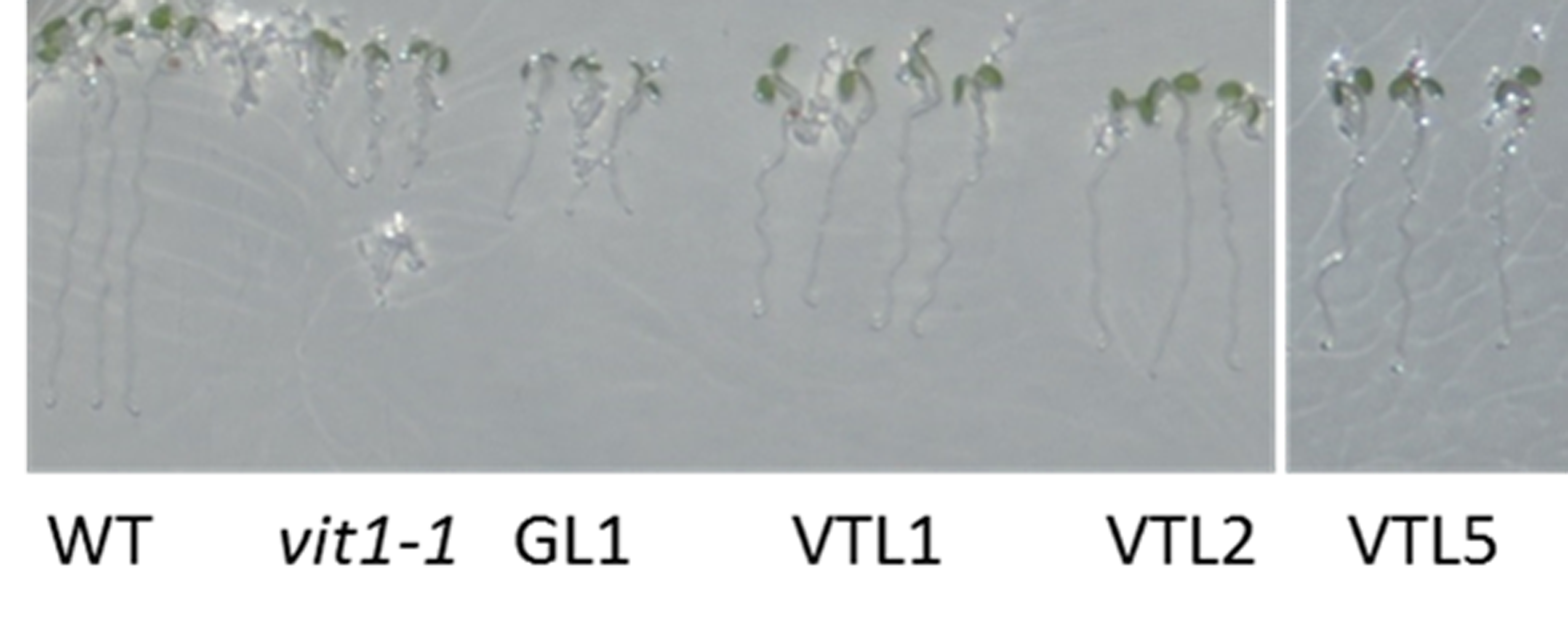

Supplement: Figure S6 — Root growth in the vit1-1 mutant transformed with AtVTL1 , AtVTL2 or AtVTL5 . Seedlings were grown for 13 days on standard media (see Materials and Methods) lacking Fe and in the presence of the Fe2+ chelator, Ferrozine. Shown are results from an experiment similar to that reported in Fig. 6. (TIF) [file pone.0110468.s006.tif]

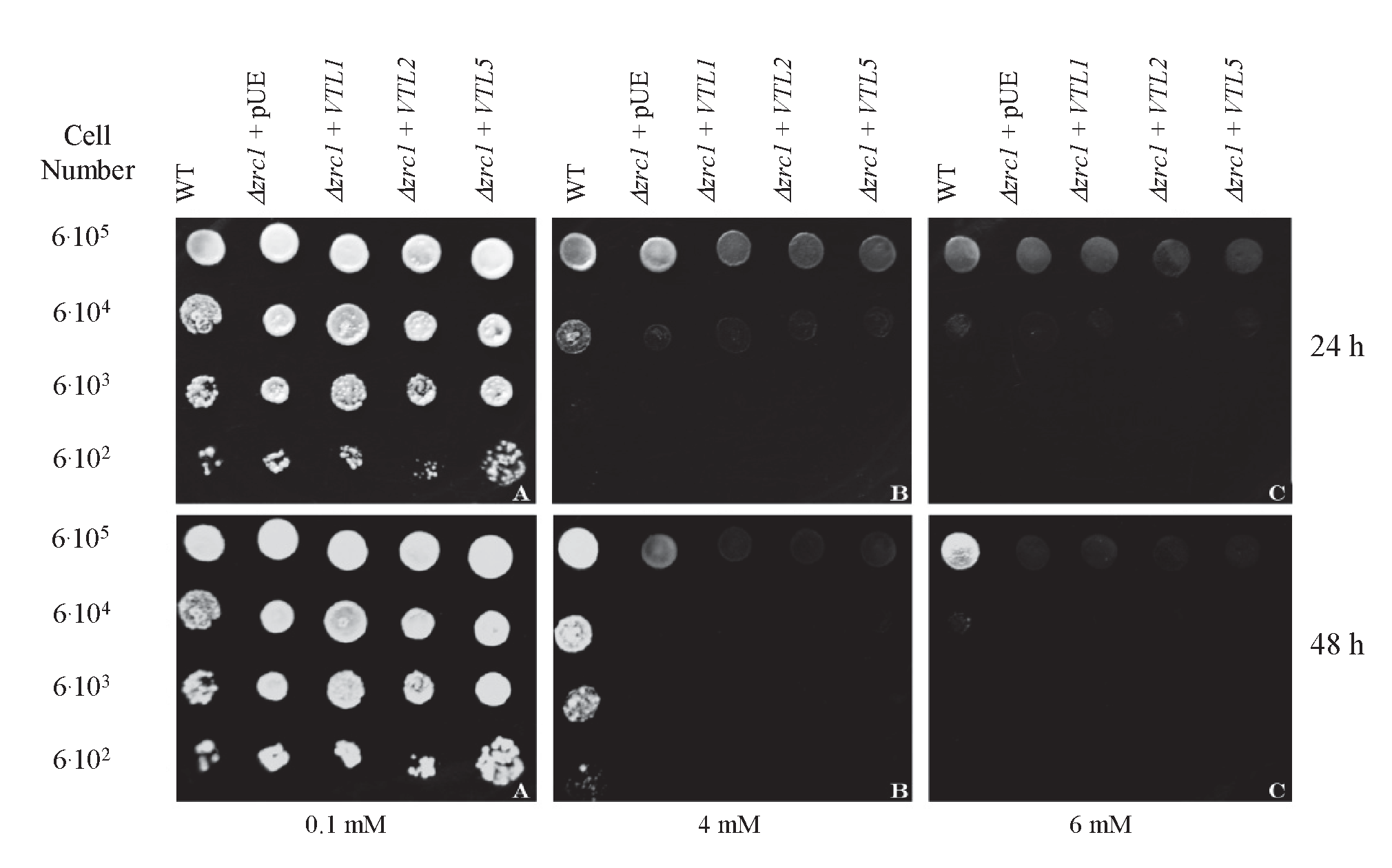

Supplement: Figure S7 — Complementation of the Δzrc1 by heterologous expression of the VTL genes. Δzrc1 (vacuolar Zn2+ transporter) cells were transformed with each of the three VTL genes or the empty vector (pUE) under the control of the PGK promoter and grown on SD medium containing ZnSO4 at the concentrations indicated for 24 or 48 h at 30°C. Cells were plated at the densities indicated in the figure. (TIF) [file pone.0110468.s007.tif]
